# Acyl-CoA thioesterase 8 induces gemcitabine resistance via regulation of lipid metabolism and antiferroptotic activity in pancreatic ductal adenocarcinoma

**DOI:** 10.1038/s41401-025-01477-y

**Published:** 2025-02-12

**Authors:** Bo-rui Li, Ting Wang, Hai-feng Hu, Di Wu, Chen-jie Zhou, Shun-rong Ji, Qi-feng Zhuo, Zheng Li, Zhi-liang Wang, Gui-xiong Fan, De-sheng Jing, Chong-yuan Yu, Yi Qin, Xue-min Chen, Jun-feng Xu, Xiao-wu Xu

**Affiliations:** 1 Department of Pancreatic Surgery, Fudan University Shanghai Cancer Center, Shanghai, 200032 China; 2 Department of Hepatopancreatobiliary Surgery, First College of Clinical Medical Science, Three Gorges University, Yichang, 443003 China; 3 People’s Hospital of China Three Gorges University, Yichang, 443099 China; 4 Department of Oncology, Shanghai Medical College, Fudan University, Shanghai, 200032 China; 5 Shanghai Pancreatic Cancer Institute, Shanghai, 200032 China; 6 Pancreatic Cancer Institute, Fudan University, Shanghai, 200032 China; 7 Department of General Surgery, First Affiliated Hospital of USTC, Hefei, 230001 China; 8 Department of Hepatopancreatobiliary, Third Affiliated Hospital of Soochow University, Changzhou, 213000 China

## Abstract

Pancreatic ductal adenocarcinoma (PDAC) comprises a group of highly malignant tumors of the pancreas. Metabolic reprogramming in tumors plays a pivotal role in promoting cancer progression. However, little is known about the metabolic alterations in tumors that drive cancer drug resistance in patients with PDAC. Here, we identified acyl-CoA thioesterase 8 (ACOT8) as a key player in driving PDAC gemcitabine (GEM) resistance. The expression of ACOT8 is significantly upregulated in GEM-resistant PDAC tissues and is closely associated with poor survival in patients with PDAC. Gain- and loss-of-function studies have shown that ACOT8 drives PDAC GEM resistance both in vitro and in vivo. Mechanistically, ACOT8 regulates cellular cholesterol ester (CE) levels, decreases the levels of phosphatidylethanolamines (PEs) that bind to polyunsaturated fatty acids and promote peroxisome activation. The knockdown of ACOT8 promotes ferroptosis and increases the chemosensitivity of tumors to GEM by inducing ferroptosis-associated pathway activation in PDAC cell lines. The combination of orlistat, an ACOT8 inhibitor, and GEM significantly inhibited tumor growth in PDAC organoid and mouse models. This study reveals the biological importance of ACOT8 and provides a potential combination therapy for treating patients with advanced GEM-resistant PDAC.

## Introduction

Pancreatic cancer, a highly heterogeneous tumor of the pancreas, is characterized by high malignancy and mortality rates. More than 90% of pancreatic cancer cases are pancreatic ductal adenocarcinoma (PDAC), which arises from the exocrine cells of the pancreas and produces digestive enzymes. Among U.S. cancer-related deaths, PDAC represents one of the top 10 causes [1], and it is expected to become the second leading cause of death by 2030 [2]. The number of patients with PDAC in China has increased, and surgical resection is the major treatment, but fewer than 20% of patients are eligible for surgery. Recently, extensive research and advanced clinical diagnosis and treatment of PDAC have notably advanced, but the 5-year survival rate of patients with PDAC has not significantly improved. Limited effective chemotherapeutic options are available for PDAC treatment, and gemcitabine (GEM) is still the major first-in-class chemotherapeutic agent in the clinical treatment of patients with advanced PDAC; however, it is common for patients with gemcitabine resistance to develop resistance after receiving active treatment for a few weeks [3]. Therefore, the identification and development of effective drugs for the treatment of GEM-resistant PDAC patients is of clinical importance.

Cancer-associated metabolic reprogramming refers to a series of alterations in extracellular and intracellular metabolites, including glycolysis, fatty acid metabolism, amino acid metabolism, oxidative phosphorylation, and redox metabolism, that occur during tumor proliferation and progression. It plays an important role in gene expression and regulation, cellular biosynthesis, and tumor microenvironment remodeling, which result in the maintenance and evolution of the tumorigenic state. Acetyl-CoA is an important intermediate metabolite from the production of catabolic reactions. It is not only used as a precursor of lipid biosynthesis but also as a substrate for acetylation reactions that regulate dynamic acetylation and the structure of histones. Homeostasis of the cellular acetyl-CoA level is required for the regulation of a variety of cellular processes, including cell growth and mitosis, transcription/translation control, and metabolism. In mammalian cells, acetyl-CoA is generated by two major catalytic enzymes, acetyl-CoA synthetase 1/2 (ACSS1/2) and adenosine triphosphate (ATP)-citrate lyase (ACLY), which are reported to play important roles in tumor progression. In addition, acetyl-CoA is metabolized exclusively by the hydrolytic enzyme acyl-CoA thioesterase (ACOT). Among this family, acyl-CoA thioesterase 8 (ACOT8) has been shown to be crucially involved in peroxisome function and mammalian lipid metabolism [4–6]. It can hydrolyze activated fatty acids to free fatty acids and CoASH [7]. Furthermore, ACOT8 was found to promote tumor development in lung and liver cancers [8, 9]. Two studies also revealed that ACOT8 was closely associated with cellular ferroptosis in urological tumors [10, 11]. However, its roles in the development of PDAC and GEM resistance are still largely unknown.

Ferroptosis, a novel form of iron-dependent cell death [12–14], is characterized by lipid peroxidation. Crucial ferroptosis-related molecules and complexes include p53, voltage-dependent anion channels, cystine/glutamate antiporters (heterodimers composed of SLC7A11 and SLC3A2), and glutathione peroxidase (especially GPX4). Recent studies have shown that ferroptosis is the critical nexus between metabolism and tumor progression. Notably, ferroptosis induction has been shown to influence the effectiveness of several conventional cancer treatments [15, 16], indicating that ferroptosis is a potential target for overcoming tumor chemoresistance[14, 17, 18]. Modulation of the expression of key molecules in the ferroptosis pathway to induce ferroptosis or reduce ferroptosis suppression may be an effective treatment option for patients with gemcitabine-resistant PDAC [17, 19]. Specifically, in our previous study, we reported that SMAD4 can bind to the promoter of GPX4 and inhibit GPX4 transcription in PDAC. TGF-β1 increased tumor sensitivity to the ferroptosis inducer RAS-selective lethal 3 (RSL3) via the SMAD4-GPX4 axis in SMAD4-positive organoids and pancreas xenograft models. Importantly, SMAD4 enhanced combination therapy with GEM and RSL3 in highly invasive PDAC cells. Ferroptosis is a complex, multistep process that is regulated by a multifaceted network in different temporal and spatial cellular contexts. The regulatory mechanism of ferroptosis by acetyl-CoA metabolic reprogramming via ACOT8 in PDAC remains elusive. Therefore, understanding the mechanisms of ferroptosis is of great importance in the development of effective strategies to reduce GEM resistance.

In this study, we further identified ACOT8 as a key player in PDAC progression, investigated its critical roles in acetyl-CoA metabolism and GEM resistance, and revealed its potential value as a new therapeutic target for combination therapy in patients with advanced GEM-resistant PDAC.

## Materials and methods

### Chemicals

Selleckchem provided the ferroptosis inducers RSL3, erastin, gemcitabine, and orlistat. MCE provided the ferroptosis inducer hemin.

### Cell culture

Our study used the PANC-1, MIA PaCa-2 and AsPC-1 cell lines from the ATCC. AsPC-1 cells were cultured in 90% RPMI-1640 medium supplemented with 10% FBS. Both PANC-1 and MIA PaCa-2 cells were cultured in DMEM containing 10% FBS, while the latter required the addition of 2.5% horse serum.

### Cellular assays

To assess cell proliferation, we used CCK-8, colony formation and IC_50_ assays.

For the CCK-8 experiment, we added the assay reagents to individual wells at different time points and incubated them for 1–2 h. Readings were taken at 450 nm using a microplate reader.

For the colony formation assays, we seeded 1000 cells into a six-well plate and incubated them for 14–21 days.

To assess the IC_50_, we added graded concentrations of the drug to the cells. Next, the CCK-8 assay was used, a concentration‒response curve was plotted; finally, the IC_50_ was calculated.

### Plasmids

The pCDH-CMV-MCS-EF1-Puro vector (System Biosciences) was used to induce ACOT8 overexpression, and the pLKO.1 TRC cloning vector (Addgene plasmid number: 10878) was used to knock down ACOT8. The two target sequences used for ACOT8 knockdown were 5'-CATTGGCGCTCAACCGAATTG-3' and 5'-CCGCCTATATCTCCGACTATG-3'.

### Western blotting (WB)

After electrophoresis, blotting, and milk sealing, the membranes were treated with the appropriate antibodies, including those against ACOT8 and catalase (ABclonal), KEAP1, SLC7A11, SLC3A2, GPX4, NRF2 (Cell Signaling Technology), and ACTB (Proteintech). The membranes were subsequently treated with rabbit or mouse secondary antibodies (ABclonal). The protein bands were visualized and photographed using a chemiluminescence kit and an imaging system.

### RT‒PCR

RNA was extracted and reverse transcribed using a reagent kit (TaKaRa). RT‒PCR was used to analyze the expression of the indicated genes using the ABI 7900HT RT‒PCR system.

The primer sequences for ACOT8 used in this study were GCTGACCACTGCATGCTCTATG (forward 5ʹ-3ʹ) and AGGTCACAGCTAGGACTCCATC (reverse 5ʹ-3ʹ). The β-actin primer sequences were CACCATTGGCAATGAGCGG (forward 5’-3’) and AGGTCTTTGCGGATGTCCACGT (reverse 5’-3’).

### FACS methodology

We used commercial lipid peroxidation (Invitrogen; BODIPY 581/591 C11, D3861) and ROS probes (Beyotime; ROS Assay Kit, S0033). The cells were stained with C11-BODIPY or DCFH-DA probes. Adherent cells were digested, resuspended, and washed before being incubated with the corresponding probe for 30 min, followed by flow cytometry analysis. The fluorescence intensity was detected by a flow cytometer (Beckman Coulter).

### Malondialdehyde (MDA) assay

We lysed the cells and determined the protein concentration, which was then assayed with an MDA detection kit (Beyotime). Next, the relative MDA content was calculated.

### Clinical specimen acquisition and selection, tissue microarray (TMA) analysis, and immunohistochemistry (IHC)

Tissue samples were obtained from PDAC patients at the Fudan University Shanghai Cancer Control Center (FUSCC). Written informed consent was obtained from each patient. This study was conducted in accordance with the ethical guidelines of the Declaration of Helsinki and was approved by the FUSCC. We included 61 patients who underwent preoperative neoadjuvant therapy at our center between June 1, 2020, and December 30, 2022. The following patients were excluded from the study: patients who did not receive a gemcitabine-based neoadjuvant; patients for whom imaging and oncology indices before and after neoadjuvant therapy could not be obtained; patients for whom data regarding the chemotherapy cycle could not be obtained; and patients whose neoadjuvant chemotherapy was completed > 8 weeks before surgery [20]. IHC was performed according to a standardized process. To quantify the results, the expression levels were categorized from weak to strong according to the staining intensity (1 to 10).

### Animal experiments

Animal experiments were conducted at the FUSCC Animal Experimentation Center under supervision.

The IACUC number for this study was FUSCC-IACUC-2022309. Mice were injected with 500,000 tumor cells in the axilla, followed by examination for tumor formation at the injection site after 2 weeks.

### Transcriptome sequencing

Transcriptome sequencing and analysis of specimens obtained from 28 patients who underwent neoadjuvant therapy were performed using Novogene. Furthermore, transcriptome sequencing and analysis of ACOT8-overexpressing and ACOT8-knockdown cell lines were performed using PANOMIX (https://www.panomix.com/). Raw data (raw reads) in fastq format were first processed through in-house Perl scripts. In this step, clean data (clean reads) were obtained by removing reads containing adapters, reads containing poly-N sequences and low-quality reads from the raw data. Moreover, the Q20, Q30 and GC contents of the clean data were calculated. All the downstream analyses were based on high-quality, clean data. The reference genome and gene model annotation files were downloaded directly from the genome website, and HISAT2 v2.0.5 was used to construct the reference genome index and compare the paired-end clean reads to the reference genome. We chose HISAT2 as the comparison tool because HISAT2 can generate splice junction databases on the basis of gene model annotation files, thus obtaining better comparison results than other nonsplice comparison tools. FPKM, the expected number of fragments per kilobase of transcript sequences per million base pairs sequenced, takes into account both the sequencing depth and gene length and is the most commonly used method to estimate gene expression levels, considering the effects of sequencing depth and gene length on read counts. Statistical routines for both were provided by the DESeq2 R package (1.20.0), which uses a model based on the negative binomial distribution to determine differential expression in numerical gene expression data and adjusts the *P* values to control for false discovery rates using the Benjamini and Hochberg method. Gene Ontology (GO) enrichment analysis of differentially expressed genes was implemented by the clusterProfiler R software package with correction for gene length bias, and GO terms with corrected *P* values less than 0.05 were considered to indicate significant enrichment of differentially expressed genes. KEGG is a database resource for understanding the high-level function and utility of biological systems, such as cells, organisms, and ecosystems, from molecular-level information, particularly large-scale molecular datasets (http://www.genome.jp/kegg/) generated by genome sequencing and other high-throughput experimental techniques. We examined the statistical enrichment of differentially expressed genes in KEGG pathways using the clusterProfiler R package. The Reactome database includes a wide range of responses and biological pathways from human model species. The DO (Disease Ontology) database describes the functions of human genes and diseases, and DO pathways with corrected *P* values of less than 0.05 were considered to indicate significant enrichment of differentially expressed genes. Gene set enrichment analysis (GSEA) is a computational method for determining whether a predefined genome is significantly and consistently different between two biological states. Genes are ranked according to their degree of differential expression in the two samples and then tested to determine whether the predefined genome is enriched at the top or bottom of the list. We used a local version of the GSEA tool http://www.broadinstitute.org/gsea/index.jsp for independent GSEA of the GO, KEGG, Reactome, DO and DisGeNET datasets. SNP calls were made using GATK (v4.1.1.0) software. The GATK standard filtering method for raw vcf files was used (other: FS > 30.0; DP < 10). Alternative splicing is an important mechanism for regulating the expression of genes and proteins. rMATS (4.1.0) software was used to analyze AS events. PPI analysis of differentially expressed genes was performed via the STRING database, which includes known and predicted protein‒protein interactions. Weighted correlation network analysis (WGCNA) is a systematic biological method for characterizing patterns of gene associations between different samples and can be used to identify highly synergistically varying sets of genes and to identify candidate biomarkers or therapeutic targets on the basis of gene set concordance and correlation between gene sets and phenotypes. The WGCNA package is a set of functions for calculating a wide variety of weighted correlation analyses, which can be used for network construction, gene screening, gene cluster identification, topological characterization, data simulation, and visualization. WGCNA is suitable for multisample data analysis, which typically requires more than 15 samples. One input file contains the sample information, i.e., the matrix describing the traits of the sample. Traits for association analysis must be numeric variables; if they are regional or categorical variables, they need to be converted to a 0–1 matrix. The other input file contains gene expression data; for transcriptome sequencing, FPKM data can be used as gene expression data.

### Nontargeted lipid metabolome sequencing

Sequencing of the untargeted lipid metabolome of ACOT8-knockdown cell lines and controls was performed using PANOMIX (https://www.panomix.com/).

LC‒MS-grade isopropyl alcohol (IPA) was purchased from Fisher Scientific (Loughborough, UK). LC‒MS-grade acetonitrile (ACN) was purchased from Dikma Technologies (51 Massier Lane, USA). Formic acid was obtained from TCI (Shanghai, China). Ammonium formate was obtained from Sigma‒Aldrich (Shanghai, China). Ultrapure water was purchased from Watsons (Guangdong, China).

LC analysis was performed on an ACQUITY UPLC System (Waters, Milford, MA, USA). Mass spectrometric detection of metabolites was performed on a Q Exactive system (Thermo Fisher Scientific, USA).

Chromatographic separation was performed with an ACQUITY UPLC® BEH C18 (2.1 × 100 mm, 1.7 µm, Waters) column was maintained at 50 °C. The temperature of the autosampler was 8 °C. The gradient elution of analytes was carried out with acetonitrile:water = 60:40 (0.1% formic acid +10 mM ammonium formate) (A2) and isopropanol:acetonitrile = 90:10 (0.1% formic acid +10 mM ammonium formate) (B2) at a flow rate of 0.25 mL/min. Two microliters of each sample was injected after equilibration. Separation was conducted under the following gradient: 0–5 min, 70%–57% A2; 5–5.1 min, 57%–50% A2; 5.1–14 min, 50%–30% A2; 14–14.1 min, 30% A2; 14.1–21 min, 30%–1% A2; 21–24 min, 1% A2; 24–24.1 min, 1%–70% A2; and 24.1–28 min, 70% A2.

The ESI‒MSn experiments were performed using spray voltages of 3.5 kV and 2.5 kV in positive and negative modes, respectively. The sheath gas and auxiliary gas were set at 30 and 10 arbitrary units, respectively. The capillary temperature was 325 °C. The Orbitrap analyzer scanned over a mass range of *m*/*z* 150–2,000 for a full scan at a mass resolution of 35,000. Data-dependent acquisition (DDA) MS/MS experiments were performed with HCD scanning. The normalized collision energy was 30 eV. Dynamic exclusion was implemented to remove some unnecessary information from the MS/MS spectra.

### Organoid construction and culture

PDAC tissues were cultured in medium (K2O-TPCSA; K2 ONCOLOGY) with agitation. The subsequently obtained cells were cultured in PDO medium. Organoids grown in Matrigel were digested at passaging and resuspended in cold phosphate-buffered saline. Next, the digested mixture was treated with an appropriate amount of trypsin (K2O-TPCSA; K2 ONCOLOGY). The organoids were subsequently resuspended in Matrigel.

### The cancer genome atlas (TCGA) analysis

Data regarding ACOT8 expression in cancerous or normal tissues of patients with PDAC in the TCGA dataset (http://www.cbioportal.org/) were collected and analyzed, and box plots were drawn.

### Statistical analysis

All the statistical analyses were performed using GraphPad Prism 7.0 and SPSS (version 22.0; IBM Corporation) software. The study data were analyzed via R software (4.1.0), ImageJ (2.14.0), GraphPad Prism (10.0), and SlideViewer (2.5.0). Pearson’s chi-square test was used for categorical variables. Continuous variables were compared via the Wilcoxon rank-sum test or Student’s *t* test. Spearman analysis was used to analyze the correlation between groups. To demonstrate the differences in means among various groups, this study employed one-way ANOVA or two-way ANOVA for statistical analysis, depending on the number of groups. Survival curves were plotted using the Kaplan‒Meier (K‒M) method and compared using the log-rank test. All the data are presented as the means ± SDs of at least three independent experiments. Two-tailed Student’s *t* tests were used for pairwise comparisons unless otherwise noted. *P* values less than 0.05 were considered significant.

## Results

### ACOT8 is highly expressed in patients with gemcitabine resistance and is associated with high malignancy and a poor prognosis

**Fig. 1:**
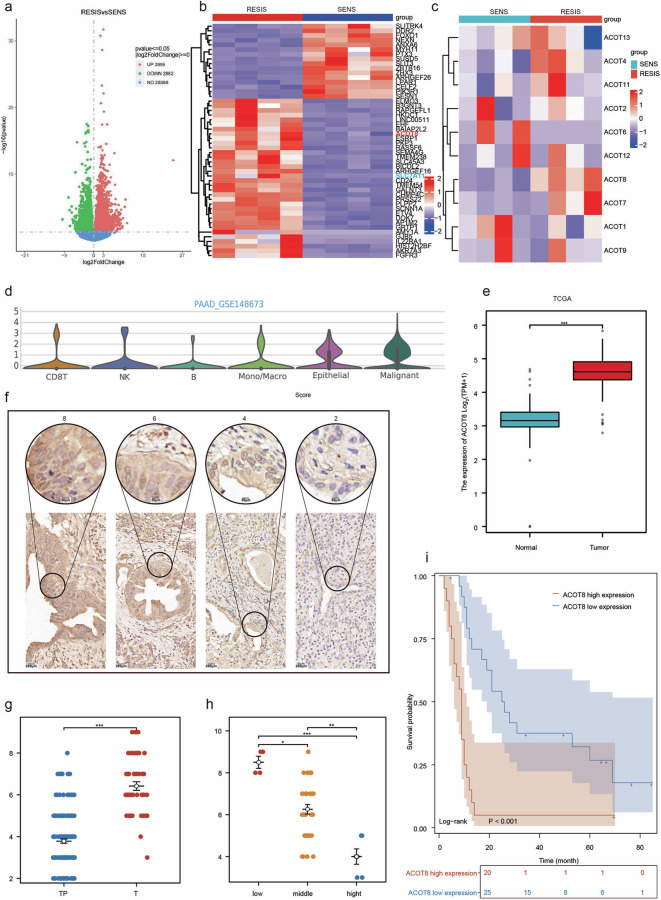
Acyl-CoA thioesterase 8 (ACOT8) is significantly overexpressed in patients with gemcitabine-resistant pancreatic ductal adenocarcinoma (PDAC) and is associated with high malignancy and a poor prognosis. **a**,** b** Differences in gene expression at the transcriptional level between the gemcitabine-resistant and gemcitabine-sensitive groups. **c** Differential expression of ACOTs between the RESIS and SENS groups. **d** Data from PAAD_GSE148673 show that ACOT8 is enriched in epithelial and malignant cells in PDAC. **e** The expression of ACOT8 in PDAC was determined via The Cancer Genome Atlas (TCGA) database. **f** Scoring of samples with different staining intensities in tissue microarrays (TMAs). **g** ACOT8 expression was significantly higher in tumor tissues (T) than in paraneoplastic tissues (TP). **h** ACOT8 expression was positively correlated with malignancy. **i** Overall survival (OS) analysis showing that patients with high ACOT8 expression levels had a poor prognosis. For data shown in this figure, statistical analyses were conducted using methods such as the t test, one-way ANOVA, and the log-rank test. *P < 0.05, **P < 0.01, ***P < 0.001.

To evaluate the alterations of the cellular metabolites in patients with GEM-resistant PDAC, we categorized patients whose serum CA199 levels decreased by >70% after neoadjuvant therapy and whose target lesion diameters, as determined by computed tomography (CT), decreased by >30% into the sensitive (SENS) group and patients whose serum CA199 levels decreased by <20% after neoadjuvant therapy and whose target lesion diameters on CT decreased by <20% into the resistant (RESIS) group [21]. Finally, 4 patients each were included in the SENS and RESIS groups. PDAC tissues were subjected to transcriptome sequencing and analysis to assess the differences in the levels of cellular metabolites in patients with GEM-resistant PDAC. In particular, lipid metabolism and acetyl-CoA metabolism were significantly different between the RESIS and the SENS control groups (Fig. 1a, b). We further analyzed the differentially expressed genes between the two groups and found that ACOT8, which plays a key role in regulating lipid metabolism and acetyl-CoA metabolism, was significantly overexpressed in the RESIS group. However, the remaining ACOT family members did not show significant differential expression (Fig. 1c), suggesting that ACOT8 may be crucially involved in gemcitabine resistance. Analysis of the PAAD_GSE148673 dataset revealed that ACOT8 was enriched in epithelial and malignant cells in PDAC tissues (Fig. 1d), and analysis of ACOT8 expression levels in the TCGA database revealed significantly higher ACOT8 expression levels in cancer tissues than in paracancerous tissues (Fig. 1e), which further suggests that ACOT8 may be involved in PDAC development. Tissue specimens for the TMA obtained from FUSCC were used for IHC and stain intensity scoring (Fig. 1f). Analysis of the staining scores and patient data revealed that ACOT8 expression was significantly higher in the tumor group (T) than in the paracancer group (TP; Fig. 1g). Additionally, the grade of tumor differentiation was correlated with the ACOT8 expression level, and the staining scores were significantly increased in the low differentiation group (Fig. 1h). Survival analysis revealed that patients with high ACOT8 expression levels had a poor prognosis (Fig. 1i). Overall, these findings suggest a significant correlation of ACOT8 expression with cancer grade, drug resistance, and patient prognosis; moreover, they demonstrate the potential diagnostic and therapeutic utility of ACOT8.

### ACOT8 regulates PDAC development in in vivo and in vitro models

**Fig. 2:**
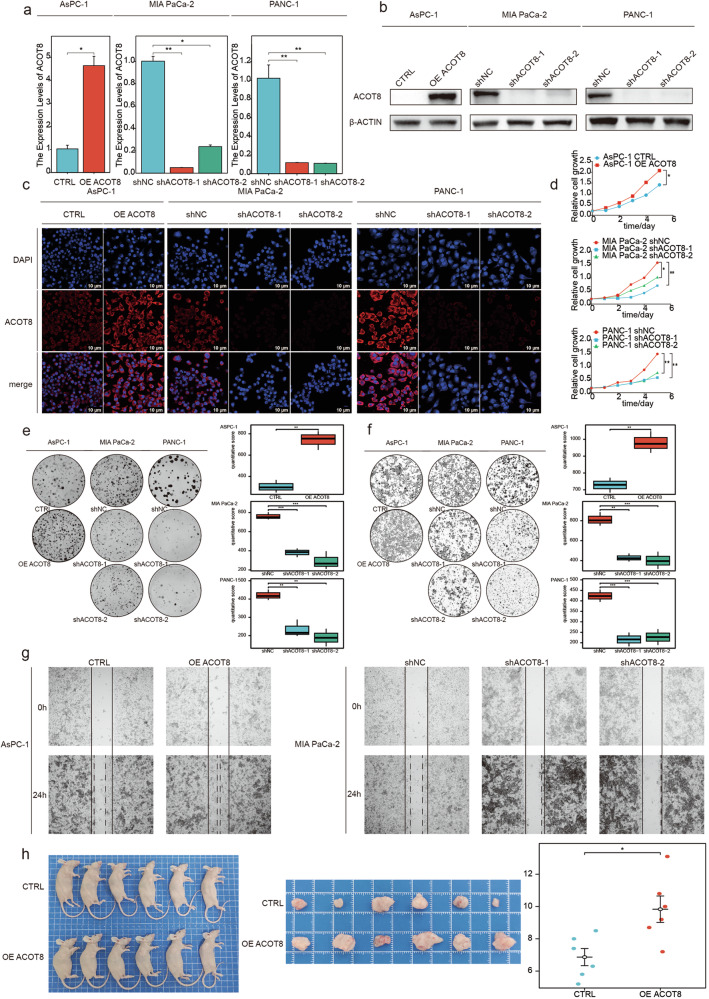
ACOT8 positively regulates the proliferative and invasive capacity of pancreatic cancer cells both in vivo and in vitro. **a**–**c** Stable ACOT8 transcripts from AsPC-1, MIA PaCa-2 and PANC-1 cells were verified via qRT‒PCR, Western blotting and immunofluorescence. **d** Effects of ACOT8 overexpression or knockdown on the proliferative capacity of PDAC cells. **e** Effects of ACOT8 overexpression or knockdown on colony formation assay results in PDAC cells. **f** Effects of ACOT8 overexpression or knockdown on Transwell assay results in PDAC cells. **g** Effects of ACOT8 overexpression or knockdown on scratch test results in PDAC cells. **h** Effects of ACOT8 overexpression or knockdown on subcutaneous PDAC cell tumor formation in nude mice. For data shown in this figure, statistical analyses were performed using methods such as the *t* test, one-way ANOVA, and two-way ANOVA. *P < 0.05, **P < 0.01, ***P < 0.001.

To investigate the role of ACOT8 in PDAC progression, we examined the expression level of ACOT8 in a variety of PDAC cell lines by WB (Supplementary Fig. 1a), from which we selected three cell lines (AsPC-1, MIA PaCa-2 and PANC-1) and constructed ACOT8 knockdown and overexpression models (Fig. 2a, b) to confirm the function of this gene in PDAC. Immunofluorescence images confirmed between-group differences in ACOT8 expression (Fig. 2c). Next, we verified the ability of ACOT8 to promote the proliferation and migration of cancer cells using cell proliferation, colony formation, Transwell, and scratch assays. We observed that ACOT8 expression levels were positively correlated with the proliferation and migration ability of cancer cells (Fig. 2d–g). Additionally, the subcutaneous tumorigenic model revealed a significantly greater tumor diameter in the ACOT8-overexpression group (Fig. 2h). Overall, ACOT8 can significantly promote the function of cancer cells; accordingly, reducing ACOT8 expression can effectively inhibit growth both in vivo and in vitro.

### ACOT8 promotes gemcitabine resistance in PDAC

**Fig. 3:**
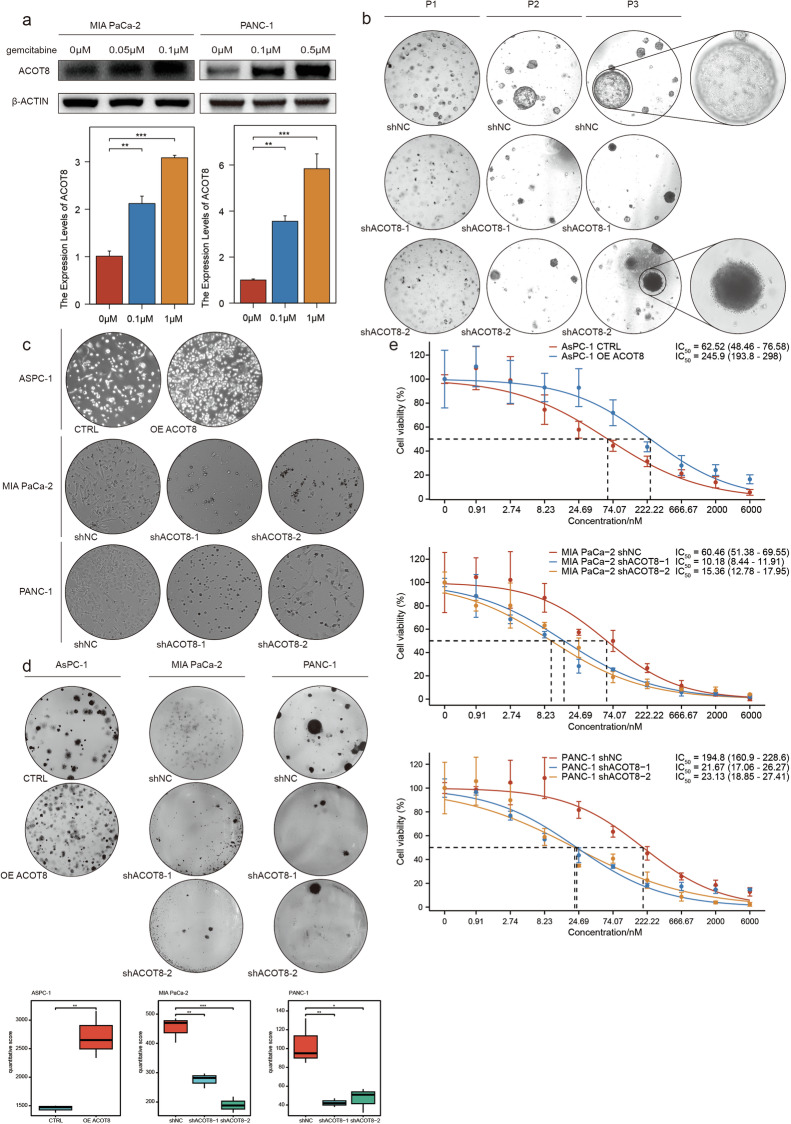
ACOT8 overexpression enhances gemcitabine resistance in pancreatic cancer cells. **a** ACOT8 expression was determined after induction by the addition of gradient gemcitabine. **b** In three different patient-derived (patient 1/2/3) organoid models, low-dose gemcitabine (20 nM) was significantly more potent in the ACOT8-knockdown group than in the control group. **c** Compared with those in the control group, the morphology of MIA PaCa-2 and PANC-1 cells in the ACOT8-knockdown group was more pronounced under the effect of gemcitabine, whereas cells in the ACOT8-overexpression group maintained a more normal morphology than cells in the control group. **d** In the colony formation assay, low-dose (30 nM) gemcitabine significantly inhibited colony formation in the ACOT8-knockdown group but weakly inhibited colony formation in the ACOT8-overexpression group. **e** Determination of the IC_50_ of gemcitabine in AsPC-1, MIA PaCa-2 and PANC-1 cells after ACOT8 overexpression or knock down. For data shown in this figure, statistical analysis was conducted via one-way ANOVA, and log-logistic analysis was employed to establish a fitting model for the drug resistance experiment. *P < 0.05, **P < 0.01, ***P < 0.001.

Treatment with a low dose of gemcitabine significantly increased ACOT8 expression in a dose-dependent manner (Fig. 3a). To elucidate whether ACOT8 levels play a role in gemcitabine resistance, we altered ACOT8 expression in organoid and cellular models. Different patient-derived PDAC organoids, especially those derived from patient 3 (P3), presented increased sensitivity to gemcitabine following ACOT8 knockdown (Fig. 3b). In the cellular models, ACOT8-knockdown cells presented a significantly greater degree of proliferation inhibition and altered cell morphology when treated with 200 and 30 nM gemcitabine (Fig. 3c, d). In contrast, in the colony formation assay, AsPC-1 ACOT8-overexpressing cells presented more clonal clusters than the controls (Fig. 3d). Finally, the ACOT8-knockdown group showed a significantly lower IC_50_ for gemcitabine than the controls (Fig. 3e). These results confirm the association between ACOT8 and gemcitabine resistance and demonstrate that ACOT8 could effectively regulate the tolerability of gemcitabine in cellular and organoid models.

### Transcriptome sequencing and ferroptosis-targeted functional assays revealed that ACOT8 regulates the ferroptosis pathway and inhibits ferroptosis in PDAC cells

**Fig. 4:**
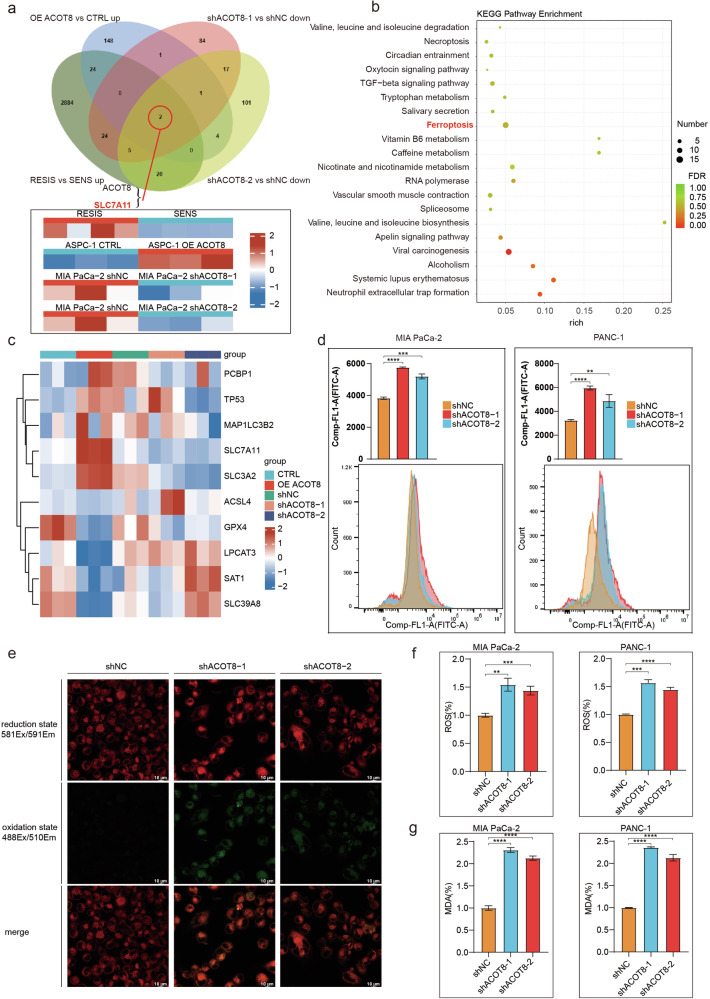
Transcriptome sequencing clustering revealed the ferroptotic pathway, and functional assays validated the inhibitory effect of ACOT8 on ferroptosis. **a** Comparisons of genes upregulated in the overexpression group, genes downregulated in the two knockout groups, and genes highly expressed in drug-resistant patients were performed, resulting in the identification of two common differentially expressed genes: ACOT8 and SLC7A11. **b** Pathway clustering of the differentially expressed genes revealed the ferroptotic pathway. **c** Differential mRNA expression of ferroptosis-related genes in different ACOT8 expression groups. **d** Detection of lipid peroxidation levels by flow cytometry in cells after ACOT8 knock down (with the ferroptosis probe BODIPY 581/591C11). **e** Detection of lipid peroxidation levels by immunofluorescence in cells after ACOT8 knock down (with the ferroptosis probe BODIPY 581/591C11). **f**,** g** Detection of ROS and MDA levels in cells after ACOT8 knock down. For data shown in this figure, statistical analysis was conducted via one-way ANOVA. **P < 0.01, ***P < 0.001, ****P < 0.0001.

To elucidate the possible mechanisms underlying the aforementioned findings, we performed transcriptome sequencing of PDAC cells in the ACOT8-overexpression and ACOT8-knockdown groups. Next, we identified overlapping genes among genes upregulated and downregulated in the ACOT8-overexpression and ACOT8-knockdown groups, respectively, as well as genes highly expressed in patients with drug resistance. Consequently, we identified two common differentially expressed genes, namely, ACOT8 and SLC7A11 (Fig. 4a). As mentioned earlier, SLC7A11 upregulation may inhibit cellular ferroptosis. KEGG pathway enrichment of differentially expressed genes identified through cellular transcriptome analysis suggested the involvement of the ferroptosis pathway (Fig. 4b). Cellular transcriptome analysis revealed that the expression of ferroptosis-promoting genes, such as *ACSL4, LPCAT3, SAT1*, and *SLC39A8*, was negatively correlated with ACOT8 expression levels. In contrast, the expression levels of ferroptosis-inhibiting genes, including *SLC7A11, SLC3A2*, and *PCBP1*, were positively correlated with ACOT8 expression levels [22, 23] (Fig. 4c). The alterations in the expression of these genes were consistent with the results of the TCGA database analysis (Supplementary Fig. 1b), which further demonstrated that elevated ACOT8 expression inhibited ferroptosis. To further determine the effect of altered ACOT8 expression on the cellular ferroptotic phenotype, we performed a C11-BODIPY assay and observed ferroptosis promotion in ACOT8-knockdown cells using flow cytometry and confocal microscopy. A significant increase in the fluorescence intensity of fluorescein isothiocyanate, which represents the level of lipid peroxidation, was noted, with the peak shifting to the right in the ACOT8-knockdown group (Fig. 4d). Furthermore, the intensity of green fluorescence, which represents the lipid peroxidation site, was greater in the ACOT8-knockdown group (Fig. 4e). Finally, we investigated the levels of ROS and MDA, important indicators of intracellular lipid peroxidation. The results revealed that both the ROS and MDA levels were significantly increased in the ACOT8-knockdown group (Fig. 4f, g). Overall, these findings indicate that ACOT8 expression is negatively correlated with ferroptosis in PDAC cells. Additionally, these genetic and phenotypic alterations involve diverse ferroptotic regulatory pathways, suggesting that ACOT8 may affect ferroptosis through diverse mechanisms.

### Inducing ferroptosis in PDAC cells reduces gemcitabine resistance caused by increased ACOT8 expression

**Fig. 5:**
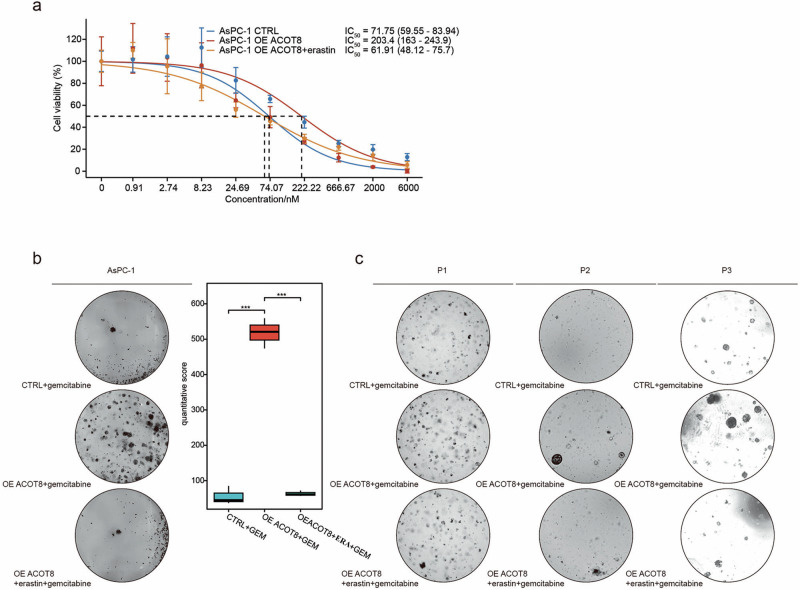
Inducing ferroptosis in pancreatic cancer cells reduces the degree of gemcitabine resistance caused by high ACOT8 expression. **a**–**c** Erastin (ERA, 3 μM) reversed the gemcitabine resistance caused by ACOT8 overexpression. For data shown in this figure, log-logistic analysis was employed to establish a fitting model for the drug resistance experiment. ***P < 0.001.

To explore the possible correlation between the inhibitory effect of ACOT8 on ferroptosis and gemcitabine resistance, we combined gemcitabine and erastin. The IC_50_ assay revealed significantly decreased resistance to gemcitabine when it was combined with low-dose erastin (Fig. 5a). Moreover, the results revealed that ACOT8-expressing cells and organoid models exhibited significant growth inhibition (Fig. 5b, c). Overall, the above results suggest that the antiferroptotic role of ACOT8 is a key factor contributing to gemcitabine resistance.

### Exploration of the mechanisms through which ACOT8 regulates ferroptosis in pancreatic cancer cells: expression of core ferroptosis-related genes, cholesterol ester and polyunsaturated phosphatidylethanolamine (PE) metabolism, and peroxisome function

**Fig. 6:**
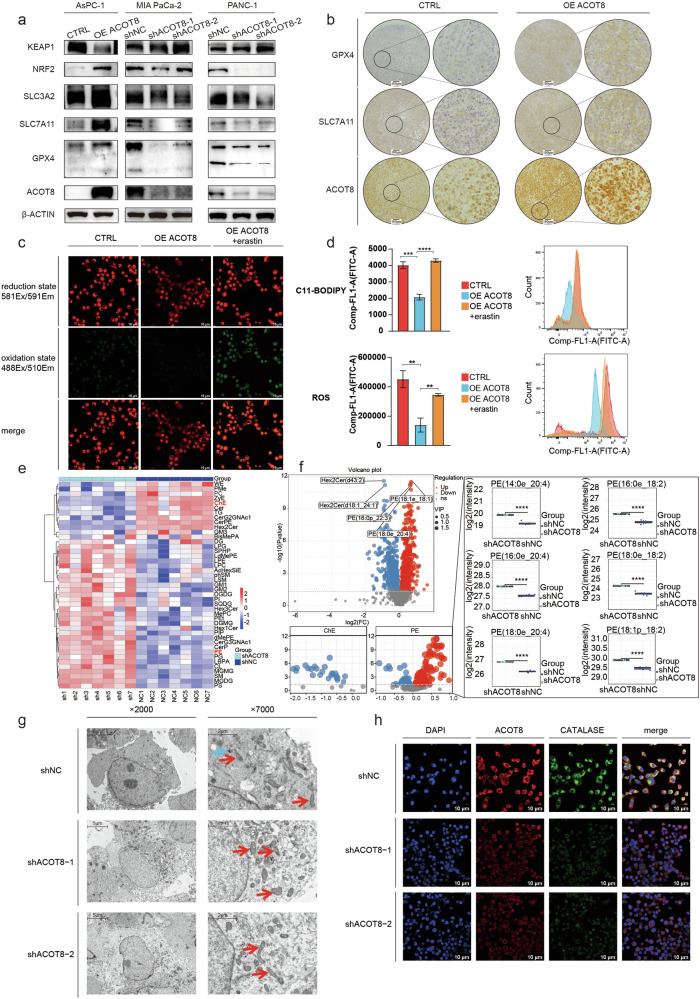
Acyl-CoA thioesterase 8 (ACOT8) inhibits ferroptosis by regulating the expression of core ferroptosis-related genes, the metabolism of cholesterol and polyunsaturated phosphatidylethanolamine (PE), and the number and function of peroxisomes. **a** Effects of altering ACOT8 expression on the expression of ferroptosis-related genes. **b** Altered expression levels of GPX4 and SLC7A11 verified by immunohistochemistry (IHC) to stain subcutaneous tumor blocks in mice. **c**, **d** Verification of the ability of 3 μM erastin to reverse ferroptosis resistance induced by ACOT8 overexpression via the ferroptosis probe BODIPY 581/591C11 and ROS detection. **e** Untargeted lipid transcriptome sequencing of MIA PaCa-2 cells after ACOT8 knock down. **f** Changes in the content and composition of cholesterol and PE in cells after ACOT8 knock down. **g** Typical ferroptosis in the mitochondria of cells after ACOT8 knock down was observed by transmission electron microscopy (red arrows: mitochondria; blue arrows: peroxisomes). **h** Altered expression of the peroxisome marker catalase in the ACOT8-knockdown group. For data shown in this figure, statistical analysis was conducted via one-way ANOVA. **P < 0.01, ***P <0.001, ****P < 0.0001.

The results of the transcriptome analysis suggested that ACOT8 may regulate ferroptosis through various mechanisms. We performed WB to verify the effect of altered ACOT8 expression on the expression levels of ferroptosis-related proteins. The findings indicated that NRF2, SLC3A2, SLC7A11, and GPX4 levels were positively correlated with ACOT8 expression levels; in contrast, KEAP1 expression levels were negatively correlated with ACOT8 expression levels (Fig. 6a), which was consistent with the inhibitory effect of ACOT8 on ferroptosis. Immunohistochemical staining of mouse subcutaneous tumors further confirmed the altered SLC7A11 and GPX4 expression levels (Fig. 6b). For further analysis, erastin was used to inhibit the Xc system, which consists of SLC7A11 and SLC3A2. Assays of cellular lipid peroxidation and ROS levels revealed that erastin reversed the inhibitory effect of ACOT8 overexpression on ferroptosis (Fig. 6c, d). These findings demonstrate that SLC7A11, a downstream molecule of ACOT8, plays a crucially role in the antiferroptotic mechanism. In addition, as ACOT8 functions as a regulatory enzyme for lipid metabolism, untargeted lipid transcriptome sequencing was conducted to determine differences in lipid metabolism between the ACOT8-knockdown and control groups (Fig. 6e). Among the observed changes, we focused on changes in the cellular CE and PE levels (Fig. 6f). We found that ACOT8 knockdown significantly decreased the intracellular levels of various CE molecules, which have been shown to play procancer roles in PDAC by participating in metabolism or regulating signaling pathways [24–26]. In contrast, the PE levels tended to increase. Moreover, ACOT8 knockdown significantly increased the levels of several PEs bound to polyunsaturated fatty acids (PUFAs), especially PEs containing 20:4 and 18:2, which are the main sites of lipid peroxidation [27]. The results revealed that after ACOT8 knockdown, the levels of multiple phosphatidylethanolamines (PEs), including 20:4 (arachidonic acid, AA) and 18:2 (linoleic acid, LA), significantly increased (Fig. 5f). The increased content of AA and LA in membrane phospholipids may render them more susceptible to lipid peroxidation [28]. During ferroptosis, PCs and PEs containing the aforementioned PUFAs are preferentially oxidized, and the resulting oxidized phospholipids are considered important death signal molecules in ferroptosis, with their accumulation being closely related to the outcome of cellular ferroptosis. These results suggest another mechanism through which ACOT8 regulates growth and ferroptosis from the perspective of lipid metabolism. To explore the typical manifestation of the antiferroptotic effect of ACOT8 at the subcellular level, we performed transmission electron microscopy examination of the cells (Fig. 6g). The mitochondria of the cells in the control group showed bulging and breakage in response to the drug, without ferroptosis. In contrast, the mitochondria of the cells in the ACOT8-knockdown group were in the typical ferroptotic stage and were partially solidified, with a slightly increased density of the membrane and intramembrane matrix; moreover, the mitochondrial cristae showed obvious expansion (red arrows in Fig. 6g). It was difficult to identify peroxisomes in cells in the ACOT8-knockdown group using electron microscopy; however, they were visible in the cells in the control group (blue arrows in Fig. 6g). According to BioGRID (https://thebiogrid.org/), ACOT8 is localized in peroxisomes and interacts with peroxisome biogenesis factor 5. To investigate whether ACOT8 expression affects peroxisomes, we performed confocal microscopy to observe the expression of the peroxisome-specific marker catalase (Fig. 6h). We observed significantly lower catalase expression in the ACOT8-knockdown group than in the control group. This indirectly reflects the regulatory effect of ACOT8 on peroxisomal function. Peroxisomes are crucial ROS-scavenging organelles that protect against ferroptosis [13]. Overall, these results suggest that ACOT8 inhibits ferroptosis by promoting the expression of antiferroptotic genes, regulating lipid metabolism, and promoting peroxisome function, which consequently results in procancer effects.

### The lipid-lowering drug orlistat reverses the inhibitory effect of ACOT8 on ferroptosis by inhibiting ACOT8 expression

**Fig. 7:**
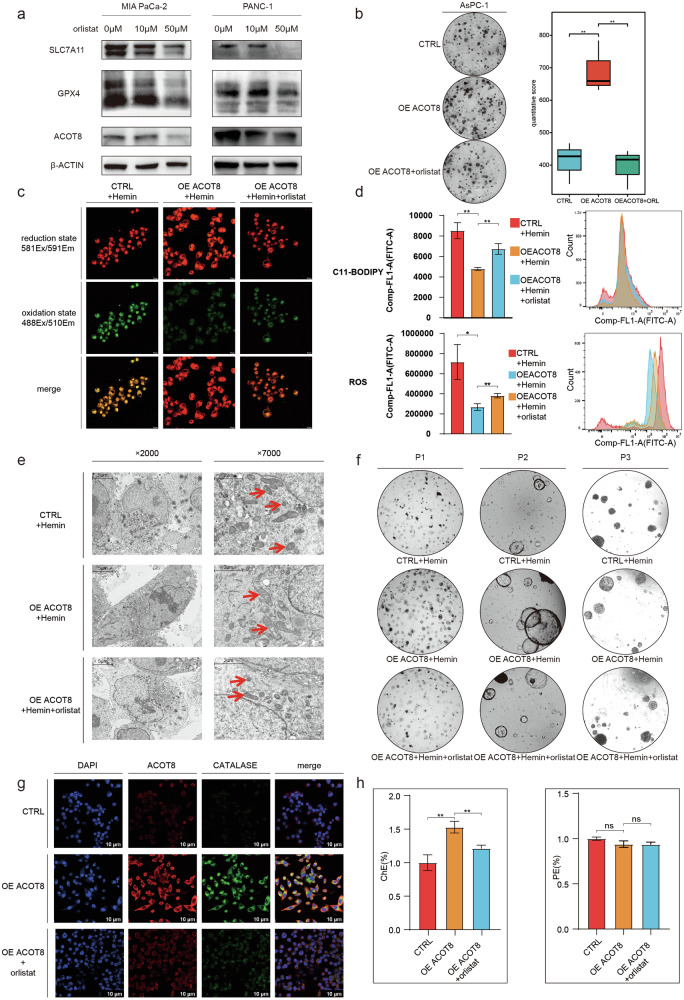
The lipid-lowering drug orlistat reverses the protective effect of acyl-CoA thioesterase 8 (ACOT8) on ferroptosis in pancreatic cancer cells by inhibiting ACOT8 expression. **a** Expression of ACOT8, SLC7A11, and GPX4 after 2 weeks of treatment of cells with graded concentrations of orlistat. **b** In the colony formation assays, 30 μM orlistat reversed the increase in AsPC-1 cell proliferation caused by ACOT8 overexpression. **c**,** d** Orlistat (30 μM in cells) reversed ferroptosis resistance caused by ACOT8 overexpression when hemin was included in the background. **e**,** f** Orlistat (30 μM in cells; 10 μM in organoids) reversed ferroptosis resistance caused by ACOT8 overexpression when hemin was included in the background. **g** Orlistat reversed catalase overexpression caused by ACOT8 overexpression. **h** Effects of orlistat on cholesterol and PE contents in the ACOT8-overexpression group. For data shown in this figure, statistical analysis was conducted via one-way ANOVA. **P < 0.01.

Orlistat, which is a lipid-lowering and weight-loss drug widely used in clinical practice, inhibits the expression of ACOT8 [29] and induces tumor cell ferroptosis [30, 31]. Therefore, we treated MIA PaCa-2 and PANC-1 cells with orlistat and performed WB after 3 days. Our findings revealed that orlistat inhibited the expression of ACOT8 as well as the ferroptosis suppressor genes SLC7A11 and GPX4 (Fig. 7a). Additionally, orlistat reversed the promoting effect of ACOT8 overexpression on colony formation (Fig. 7b). Similarly, C11-BODIPY assays, ROS assays, transmission electron microscopy, and organoid assays revealed that orlistat attenuated the inhibitory effect of ACOT8 overexpression on ferroptosis (Fig. 7c–f). To increase basal ferroptosis and accentuate between-group differences, we induced ferroptosis using hemin, which is a ferrous ion carrier that is not related to the possible mechanism under investigation. With respect to peroxisome function and lipid metabolism, orlistat similarly reversed the promotion of peroxisomal function and reduction in CE levels induced by ACOT8 (Fig. 7g, h). Overall, orlistat can inhibit ACOT8 expression and the downstream ferroptosis inhibitory pathway. Moreover, it can functionally reverse the changes caused by high ACOT8 expression in various ways; therefore, orlistat can be considered a potential ACOT8 inhibitor.

### Orlistat enhances the gemcitabine sensitivity of PDAC cells in vivo and in vitro

**Fig. 8:**
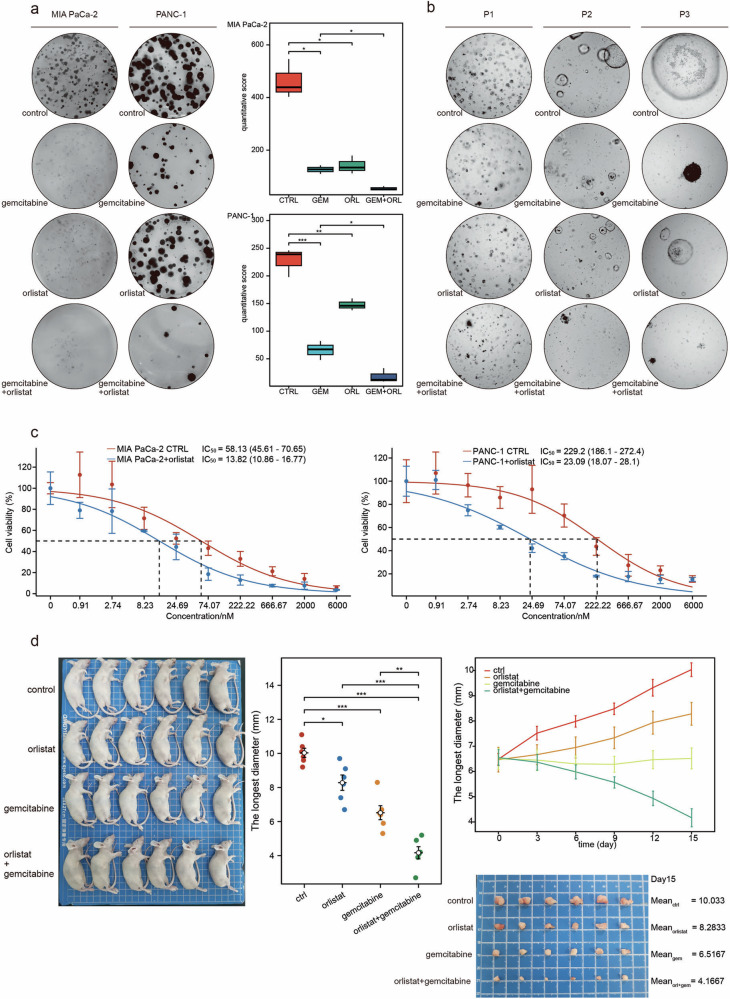
The lipid-lowering drug orlistat enhances the gemcitabine sensitivity of PDAC in vivo and in vitro. **a** Effects of 30 μM orlistat and 50 nM gemcitabine alone or in combination on MIA PaCa-2 and PANC-1 cells in colony formation assays. **b** Effects of 20 μM orlistat and 20 nM gemcitabine alone or in combination on organoids. **c** Changes in the IC_50_ of gemcitabine in combination with orlistat. **d** Effects of an intraperitoneal injection of 30 mg/kg orlistat and 10 mg/kg gemcitabine alone or in combination on subcutaneous tumor formation in nude mice. *P < 0.05, **P < 0.01, ***P < 0.001.

To further elucidate the possible mechanism underlying the ability of orlistat to promote gemcitabine sensitivity, we examined the effects of orlistat combined with gemcitabine in cellular, organoid, and nude mouse subcutaneous tumorigenic models. The findings indicated that orlistat alone had a weak inhibitory effect on PDAC; however, it significantly enhanced the inhibitory effect of gemcitabine (Fig. 8a, b). Moreover, IC_50_ assays confirmed that the coadministration of orlistat with gemcitabine significantly reduced the IC_50_ of gemcitabine in PDAC cells (Fig. 8c). For the animal experiments, we administered intraperitoneal injections of low-dose gemcitabine (30 mg/kg) or orlistat (10 mg/kg) to each group of mice at 3-day intervals. Within 15 days, the tumor diameter in the gemcitabine + orlistat group significantly decreased, exhibiting significantly greater efficacy than the other groups. After 15 days of treatment, the tumors of mice in the gemcitabine + orlistat group were significantly smaller than those of mice in the gemcitabine or orlistat groups (Fig. 8d). Overall, these findings indicate that orlistat promotes the activity of gemcitabine against PDAC and suggest its potential for reducing gemcitabine resistance.

## Discussion

Given that the vast majority of patients with PDAC miss the opportunity for surgery [32], chemotherapy is an important part of the treatment regimen, with gemcitabine being the preferred chemotherapeutic agent [33]. Although gemcitabine is effective in the early stages of its application, patients with PDAC often develop gemcitabine resistance over time, resulting in a poor prognosis [34]. Accordingly, this study aimed to identify key molecules involved in gemcitabine resistance by investigating altered gene expression levels in gemcitabine-resistant tumor tissues. Transcriptomic sequencing analysis identified the core molecule, ACOT8, as a differentially expressed gene. Notably, the downstream molecule SLC7A11, which was identified in subsequent analyses, also demonstrated consistent differential expression (Fig. 1b), further suggesting a strong connection between ACOT8 and SLC7A11 and the potential of targeting ferroptosis to overcome gemcitabine resistance. The relationship between ferroptosis and tumor therapy has been widely recognized, with many reports indicating that ferroptosis induction promotes the antitumor effect of chemotherapeutic agents [19, 34] or directly kills tumor cells [15, 35].

To determine the effects of ACOT8 on phenotype, we established ACOT8-overexpression and ACOT8-knockdown cell lines, as well as the corresponding controls. We observed that ACOT8 promoted cell proliferation, migration, and gemcitabine resistance in vivo and in vitro. Additionally, ACOT8 overexpression affected the morphology of AsPC-1 cells (Fig. 2c), which exhibit a low level of differentiation. However, under conditions of ACOT8 overexpression, these cells were more inclined to transform into shuttle-shaped cells. This change in cell morphology was stable and consistent with other experimental results (Fig. 7g). However, the mechanism underlying this phenomenon remains unclear, and determining whether this phenomenon is associated with ferroptosis is important. We speculate that this phenomenon is related to the effect of ACOT8 overexpression on lipid metabolism.

To elucidate the underlying mechanism, we determined the antiferroptotic effect of ACOT8 using transcriptome sequencing and subsequent experiments. The role of the antiferroptotic effect in chemotherapeutic agent resistance, especially gemcitabine resistance, has been widely reported [17, 19, 36]; however, the underlying mechanism remains unclear. We believe that both the role of ferroptosis in gemcitabine resistance and the mechanism through which ACOT8 regulates ferroptosis are complex and comprehensive and should not be understood only in terms of limited gene pathway regulation. We interpreted the role of ACOT8 with respect to the regulation of key genes involved in ferroptosis, alterations in lipid metabolism, and peroxisomal alterations. Although it is difficult to elucidate a mechanism that integrates these three effects, we speculate that the regulation of lipid metabolism may contribute to peroxisomal alterations. This is because cholesterol and phosphatidylcholine are crucial components of organelle membranes; additionally, their reduced levels in ACOT8-knockdown cells (Fig. 6e) may have affected the structural integrity of the intracellular membranes, which led to a reduction in the number and function of peroxisomes. Additionally, ACOT8 knockdown increased the levels of PEs bound to PUFAs (Fig. 6f), whereas the total amount of PEs was not affected by the ACOT8 expression level (Fig. 7h). These findings suggest that ACOT8 is involved in altering the proportion of PEs containing proferroptotic motifs rather than altering the total number of PEs.

Additionally, previous research findings [37] and our coimmunoprecipitation (co-IP) experiments with the ACOT8 protein both demonstrated a direct interaction between ACOT8 and PEX5. The primary role of PEX5 is to bind proteins containing peroxisomal targeting signal 1 (PTS1) and to participate in the protein import process into peroxisomes. Consequently, the entry of ACOT8 into peroxisomes is regulated by PEX5. These findings also suggest that functional alterations in PEX5 may impact the gemcitabine resistance mediated by ACOT8.

In addition, we assayed DCK1, HENT1, and RRM1, which are crucial for gemcitabine resistance in PDAC, through WB and observed no correlation of their expression with ACOT8-mediated gemcitabine resistance (Supplementary Fig. 1e). These findings indicate that ACOT8-mediated gemcitabine resistance is regulated via a novel pathway.

Our findings suggest that orlistat induces ferroptosis and overcomes gemcitabine resistance by inhibiting ACOT8 expression. Currently, the mode of administration available for orlistat does not allow it to enter the blood circulation, but it affects intestinal cells. However, the direct action of orlistat on tumor cells is known to induce ferroptosis and inhibit tumor cell growth [30, 38, 39]. Therefore, exploring alternative modes of orlistat administration may be beneficial for further clinical applications.

This article has several limitations that require further clarification in subsequent studies. Owing to the limitations of our nontargeted lipidomic detection technology, the examination of cellular cholesterol metabolism included only various cholesterol esters and did not detect changes in the content of free cholesterol. Therefore, further experiments involving more targeted detection are needed to more comprehensively elucidate the impact of ACOT8 on cholesterol metabolism. Additionally, this study struggled to elucidate the precise relationship between the enzymatic function of ACOT8 and its role in ferroptosis resistance, and it failed to identify other specific mechanisms by which ACOT8 regulates ferroptosis. We detected changes in the cellular acetyl-CoA content and histone acetylation levels, and the experimental results were consistent with the enzymatic function of ACOT8 (Supplementary Fig. 1c, d). However, the effects of ACOT8 on gene expression, lipid metabolism, peroxisome number, and function remain unclear. To further explore the mechanisms by which changes in ACOT8 expression lead to these effects, acetylation proteomic sequencing and isotopic labeling molecular pathway studies of lipid metabolism may be necessary. Finally, this study primarily discusses the protective role of ACOT8 against ferroptosis under the stress conditions caused by a chemotherapy drug (gemcitabine). These results indicate that ACOT8 can exert stress-protective effects by participating in the regulation of cellular lipid metabolism. However, the regulatory role of ACOT8 in the metabolic state of pancreatic cancer cells and the mechanism of ferroptosis under other common stress conditions (such as hypoxia) remain to be explored. Future research could focus on the metabolic regulatory effects of ACOT8 under various stress conditions, with the aim of comprehensively elucidating other potential stress-protective mechanisms of ACOT8.

**Fig. 9:**
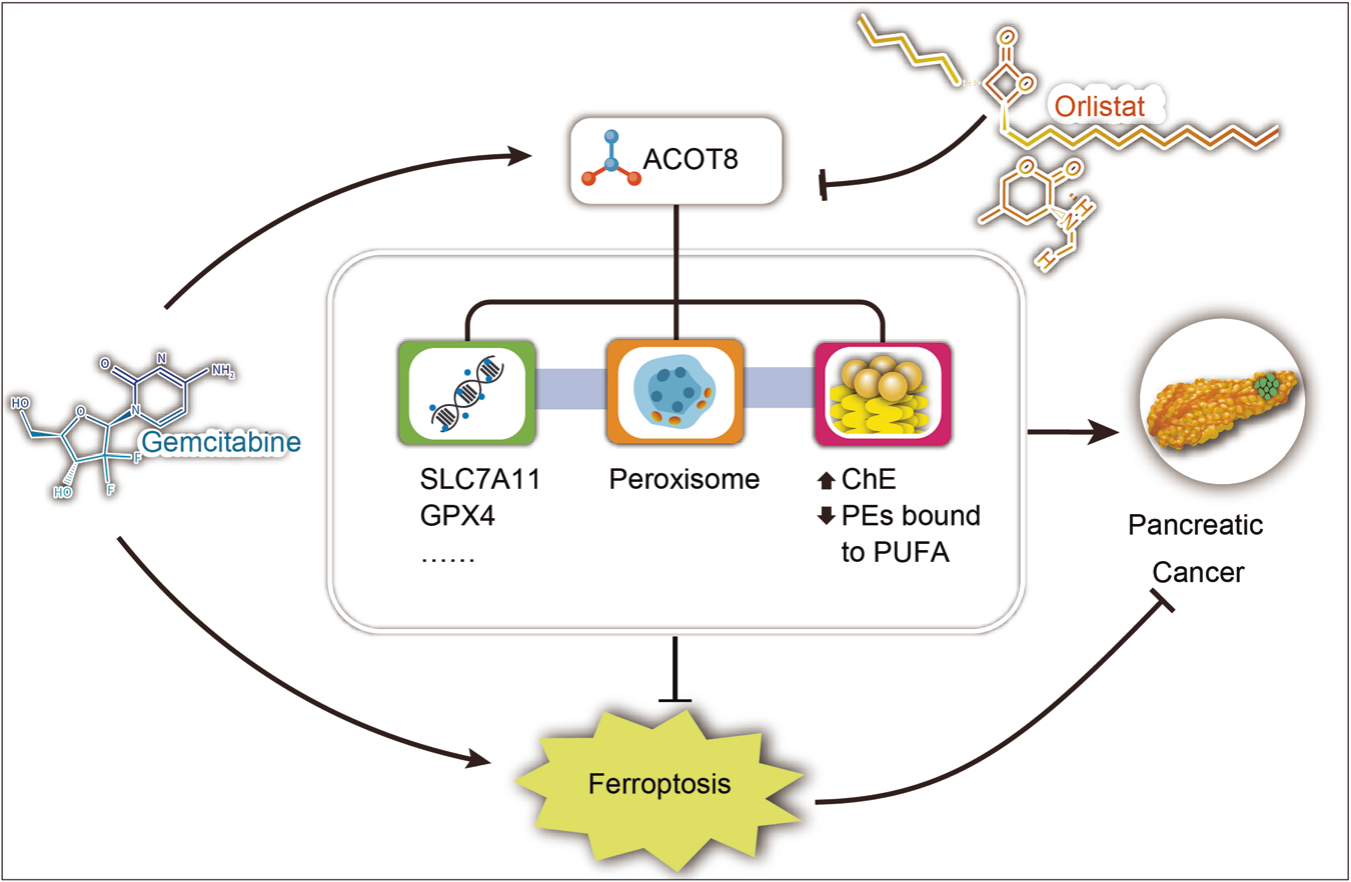
ACOT8 inhibited gemcitabine-induced pancreatic cancer cell  ferroptosis by regulating ferroptosis-related gene pathways, organelle function, and lipid metabolism, thereby inducing pancreatic cancer gemcitabine resistance.

In summary, our findings demonstrate that ACOT8 is an important molecule for gemcitabine resistance. Further studies revealed that the promoting effect of ACOT8 on gemcitabine resistance was closely related to its antiferroptotic effect. Additionally, we investigated the possible regulatory mechanisms of ACOT8 in ferroptosis from three perspectives: gene pathways, lipid metabolism, and organelle function (Fig. 9). The regulatory effect of orlistat on ACOT8 expression further demonstrates its potential clinical significance in PDAC chemotherapy. In conclusion, the combined administration of orlistat and gemcitabine may contribute to overcoming gemcitabine resistance and thus improve the prognosis of patients.

## Supplementary information

Supplementary Figure 1
