# Supplementary material for: Acyl-CoA thioesterase 8 induces gemcitabine resistance via regulation of lipid metabolism and antiferroptotic activity in pancreatic ductal adenocarcinoma
Source: Acta Pharmacol Sin. 2025 Feb 12;46(6):1742–56. doi: 10.1038/s41401-025-01477-y (PMC12098905; doi:10.1038/s41401-025-01477-y)
Supplement: Supplementary file 1 — Supplementary Figure 1 [file 41401_2025_1477_MOESM1_ESM.docx]

**
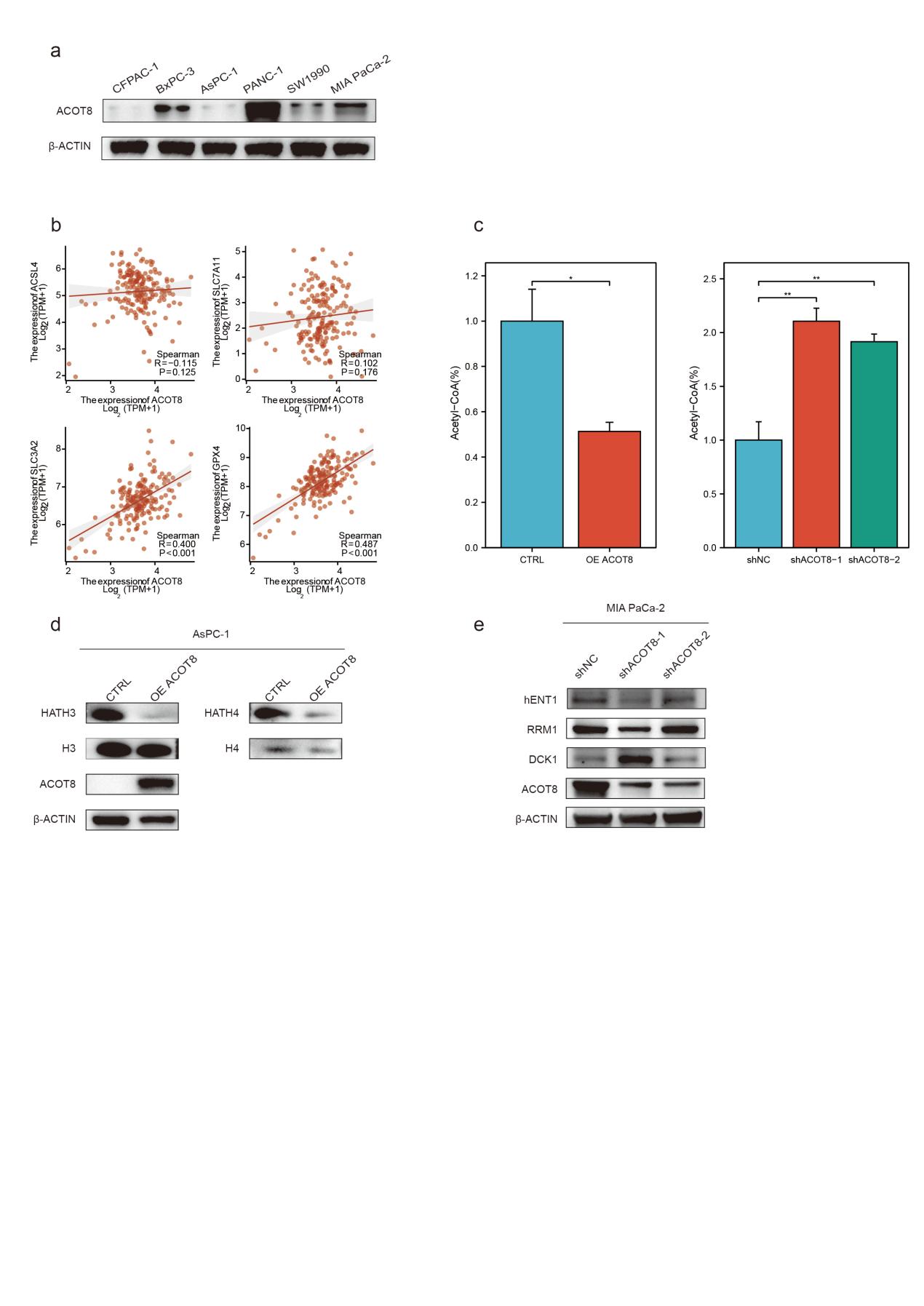
**

**Supplementary Figure 1. (a)** ACOT8 expression levels in various PDAC cell lines. **(b)** Correlation of the expression of some core ferroptosis-related genes with ACOT8 in The Cancer Genome Atlas (TCGA) database. **(c)** Altered intracellular acetyl-CoA content due to changes in ACOT8 expression levels. **(d)** Altered histone acetylation levels due to changes in ACOT8 expression levels. **(e)** Effects of altered ACOT8 expression levels on the classical gemcitabine resistance pathway.
